# LRTM effect and electronic crystal imaging on silicon surface

**DOI:** 10.1038/s41598-021-87629-6

**Published:** 2021-04-16

**Authors:** Zhong-Mei Huang, Shi-Rong Liu, Hong-yan Peng, Xin Li, Wei-Qi Huang

**Affiliations:** 1 Institute of Nanophotonic Physics, Guizhou University, Guiyang, 550025 China; 2 Department of Physics, Hainan Normal University, Haikou, 571158 China; 3 State Key Laboratory of Environment Geochemistry, Institute of Geochemistry, Chinese Academy of Sciences, Guiyang, 550003 China

**Keywords:** Applied optics, Optical physics, Environmental sciences

## Abstract

Some interesting phenomena have been observed in the laser reflecting Talbot magnification (LRTM) effect discovered at first, in which the high-order nonlinear imaging and the plasmonic structures imaging occur. The LRTM effect images were obtained on the 1D and 2D photonic crystals fabricated by using nanosecond pulsed laser etching on silicon surface, where the high-order nonlinear imaging on the 1D and 2D photonic crystals was observed interestingly. The theory result is consistent with the experimental one, which exhibits that the suitable wave-front shape of injection beam selected in optical route can effectively enlarge the magnification rate and elevate the resolution of the Talbot image. Especially the periodic plasmonic structures on silicon surface have been observed in the LRTM effect images, which have a good application in the online detection of pulsed laser etching process. The temporary reflecting Talbot images exhibit that the electrons following with photonic frequency float on plasma surface to form electronic crystal observed on silicon at first, which is similar with the Wigner crystal structure.

## Introduction

The beauty and simplicity of Talbot effect images, so-called self-imaging effect, attracted scientists and resulted in numerous interesting and applications. As first, H.F.Talbot discovered the conventional Talbot effect in 1836^1^. Then, the effect was well understood by the Fresnel-Kichhoff diffraction theory and was explained analytically by Lord Rayleigh in 1881^2^. The recent developing has been made in the areas, which has attracted extensive research interesting due to the promising physical properties concerning various applications^3–14^. Nowadays, the new phenomena and their application in the Talbot effect have provided a new way for micro-nanostructures to be observed and analyzed by simply projecting image, involving phase components and amplitude, nonlinear images, various micro-nanoarrays images and surface plasmonic structures images^15–31^. In addition, the recent progress about the bi-layer structure image in the electromagnetic field was reported^32^. And the self-imaging effect with a similar approach in atomic ensemble EIT media has been noted as well^33,34^.

In the article, the LRTM effect has been discovered on one-dimensional (1D) and two-dimensional (2D) arrays on silicon surface under the illumination of Gaussian spherical wave and plane wave, which is different from the conventional Talbot effect. It provides a new way to magnify the reflecting image for observation of thicker objects with periodic structures on surface. The 1D and 2D photonic crystals on silicon surface were fabricated by using nanosecond pulsed laser etching (PLE) method, where their high-order nonlinear images were observed in the LRTM effect interestingly. Here, the resolution of the LRTM effect image was improved due to originate from the high-order nonlinear diffraction. The magnifying image of plasmonic structures in the LRTM effect was observed on silicon surface. It is interesting that the temporary reflecting Talbot images exhibit that the electrons following with photonic frequency float on plasma surface to generate electronic crystal observed at first, which is similar with the Wigner crystal structure.

### LRTM effect

In the experimental results, the magnification rate of the LRTM effect image and the interval between two adjacent reflecting Talbot images could be controlled by adjusting wave-front shape of illumination to enlarge the magnification rate.

**Figure 1:**
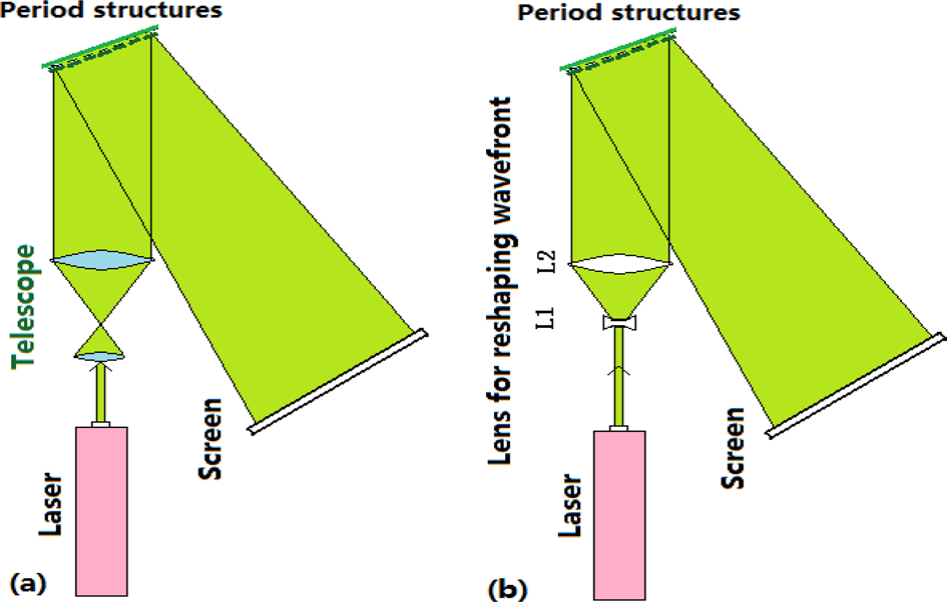
(**a**) Experimental diagram designed for reshaping Gaussian beams to plane wave, in which the telescope is used in inverse optical path. (**b**) Experimental diagram designed for enlarging spot of Gaussian beams, where the beam wave-front can be reshaped by adjusting lens L1 and L2 in the LRTM routing.

The 1D and 2D photonic crystal of silicon as the samples were fabricated by using nanosecond PLE method in room temperature. The laser beams at 532 nm or at 805 nm are as the foundation injection waves for illumination in the LRTM image, which are reshaped and then illuminated on the samples. In the image process, a telescope device along with inverse light path is used to obtained plane wave for illuminating samples, whose image is shown in Fig. 1a where the inset exhibits its diffraction pattern. In other way, we can tailor the wave-front shape of injection beam to get Gaussian spherical wave by adjusting beam spot in the LRTM image process, whose image is exhibited in Fig. 1b where the inset shows the diffraction pattern of the 2D lattice.

**Figure 2:**
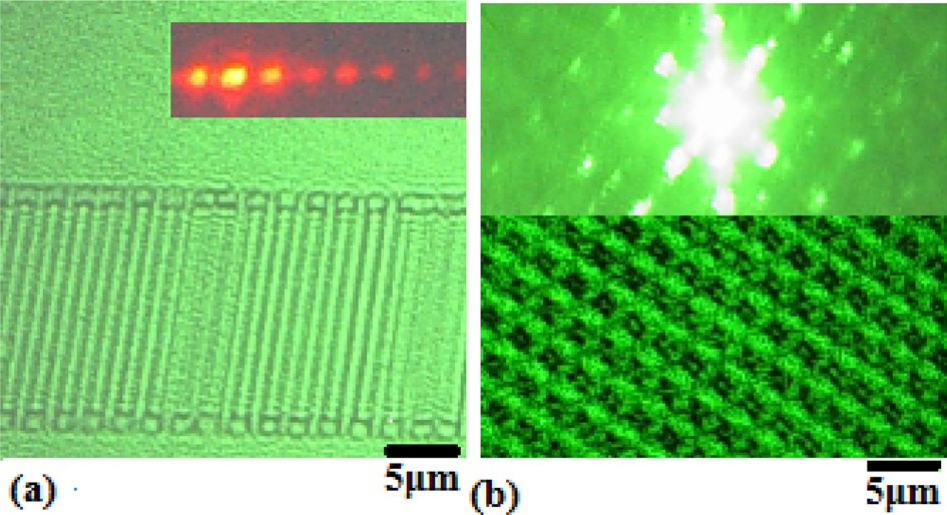
LRTM images on 1D (**a**) and 2D (**b**) photonic crystals of silicon under laser illumination, in which the inset windows show the space frequency images (diffraction patterns) of 1D and 2D photonic crystal structures.

Under the laser illumination, Fig. 2a shows the LRTM image on the 1D photonic crystals of silicon prepared by using PLE method, in which the inset window exhibits its diffraction pattern showing the space frequency of the 1D photonic crystal structure. Here, the space frequency is 1/(2 μm) and the depth is about 3 μm. In the same way, the LRTM image is observed on the 2D photonic crystals fabricated by using PLE method on silicon, as shown in Fig. 2b, where the inset exhibits the diffraction pattern of the 2D photonic crystals of silicon.

**Figure 3:**
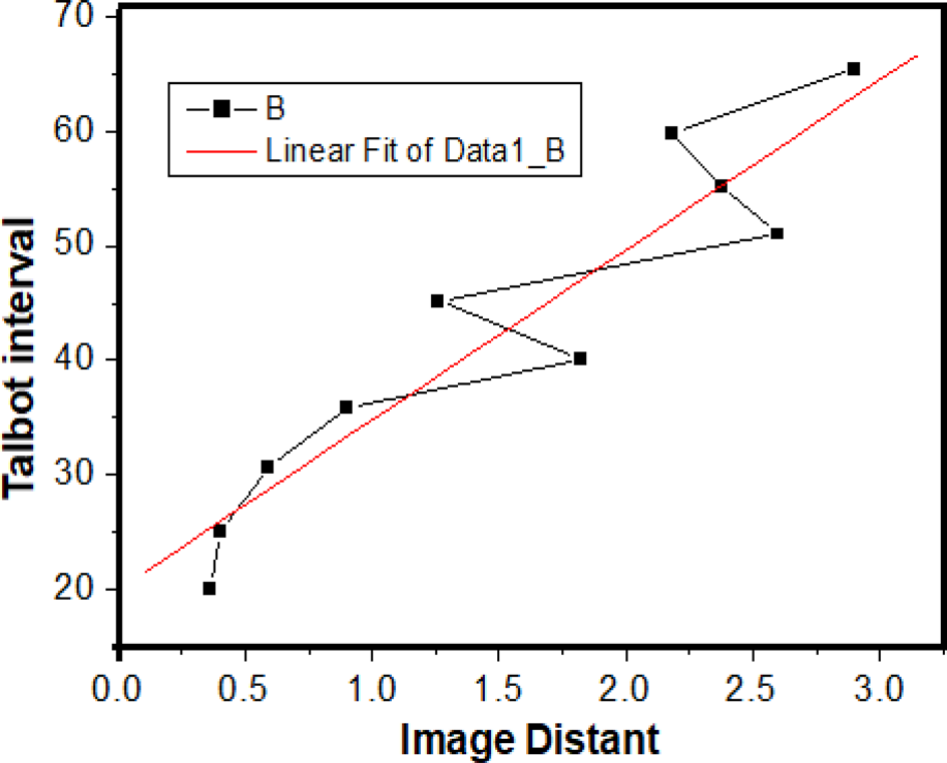
The curve of relationship between the Talbot interval and the imaging distance in the LRTM process, in which the black curve describes the experimental result and the red line is linear fit one.

The experimental results exhibit the relation between the interval of adjacent images so-called the Talbot interval and the imaging distance so-called the Talbot distance, as shown in Table  1, which displays an almost linear increasing evolution in the LRTM effect. The relationship between the Talbot interval and the imaging distance is shown in Fig. 3, where the red line is linear fit one and the black curve describes the experimental result in the LRTM process. In the detection, the spatial constant of periodic structure is 50 μm, and the laser beam at 532 nm is used as the Talbot beam. The linear rates can be controlled by adjusting wave-front shape for illumination. It is found that the magnifying rate of reflecting Talbot images is enlarged with increase of image distance. The magnifying rate can be enlarged by decreasing curvature radius R_1_ of the wave-front, which is important for application of microscopy on reflection samples.

**Table 1:** Relation between the interval of adjacent images and the imaging distance in the LRTM image process.

Talbot interval	0.36	0.4	0.59	0.9	1.82	1.26	2.6	2.38	2.18	2.9
Image distance	20.04	25.05	30.68	35.82	40.1	45.18	51	55.06	59.8	65.42

Unit/mm.

The Talbot effect belongs to a kind of transparent image, and the new reflecting Talbot image equals to the transformation of its optical path, in which it needs to change **π** phase of propagating wave-front on surface according to the Maxwell theory on electromagnetic wave^15,16^. The reflecting Talbot image amplitude A(r) can be described by the formula:

1 $$ {\text{A}}\left( {\text{r}} \right) \, = \sum_{{\text{n}}} {\text{A}}_{{\text{n}}} {\text{exp}}\left[ {{\text{i}}\left( {\uppi \uplambda {\text{n}}^{{2}} {\text{R}}_{{1}} {\text{r}}_{{2}} } \right)/\left( {\left( {{\text{ R}}_{{1}} + {\text{ r}}_{{2}} } \right){\text{d}}^{{2}} } \right)} \right]{\text{exp}}\left[ { - {\text{i}}\left( {{2}\uppi {\text{nR}}_{{1}} {\text{r}}} \right)/\left( {\left( {{\text{ R}}_{{1}} + {\text{ r}}_{{2}} } \right){\text{d}}} \right)} \right] $$

In the form, d is the grating constant of periodic structures on surface, R_1_ is the curvature radius of the Gaussian wave-front and r_2_ is the propagating distance from the reflection object to the observation screen. The reflecting Talbot image localizes at r_2_ = r_m_ and its phase term can be described by the form:

2 $$ \left[ {\uppi \left( {\uplambda {\text{n}}^{{2}} {\text{R}}_{{1}} {\text{r}}_{{2}} } \right)/\left( {\left( {{\text{ R}}_{{1}} + {\text{ r}}_{{2}} } \right){\text{d}}^{{2}} } \right)} \right]  = 2{\text{ m}}\uppi $$

where the m is a grade number of the reflection Talbot image. Therefore, the condition for reflecting Talbot imaging is r_m_ = mβ/[1 − (mβ)/R_1_], where β is (2 d^2^)/λ. It should be noted that there is r_m_ ≈ mβ[1 + (mβ)/R_1_] and the magnifying rate of the reflecting Talbot image is M_m_ = 1 + r_m_/R_1,_ when R_1_ >  > β. When the lens for magnifying in the experimental instruments are replaced by the telescope device, the R_1_ will turn to infinity and the distance r_m_ of the reflecting Talbot imaging will be mβ related to illumination of plane wave, here the interval distance of reflecting Talbot images is almost a constant.

**Figure 4:**
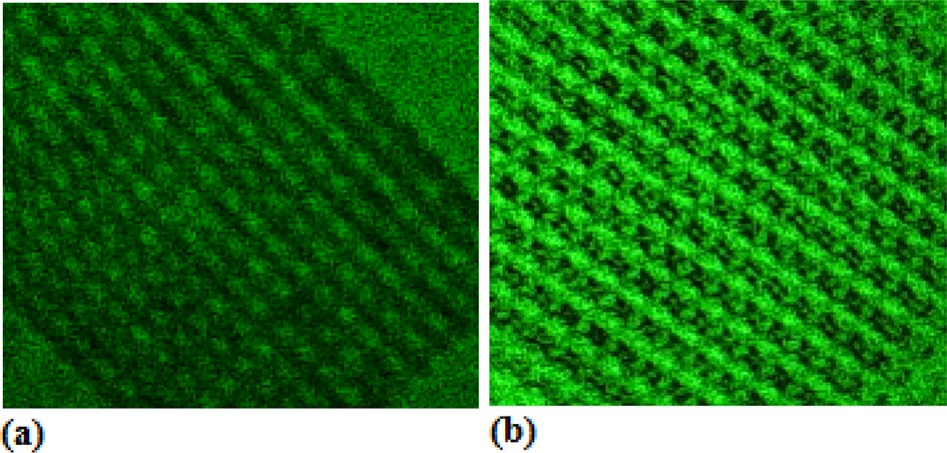
(**a**) Dark field image and (**b**) Bright field image, which appear at r_2_ = r_m_ and r_2_ = r_m_ + Δr respectively , where the Δr relates to the phase term [π(λn^2^ R_1_ r_2_)/(( R_1_ + r_2_)d^2^)] = (2 N + 1)π.

These results are consistent with that of experiments. It is interesting that the bright field image or the dark field image appears at r_2_ = r_m_ or r_2_ = r_m_ + Δr, respectively, where the Δr relates to the phase term [π(λn^2^ R_1_ r_2_)/(( R_1_ + r_2_)d^2^)] = (2 N + 1)π, as shown in Fig. 4a (dark field image) and in Fig. 4b (bright field image). The magnifying rate of the LRTM measured in the experiments reaches to about 2000.

### Nonlinear LRTM images

**Figure 5:**
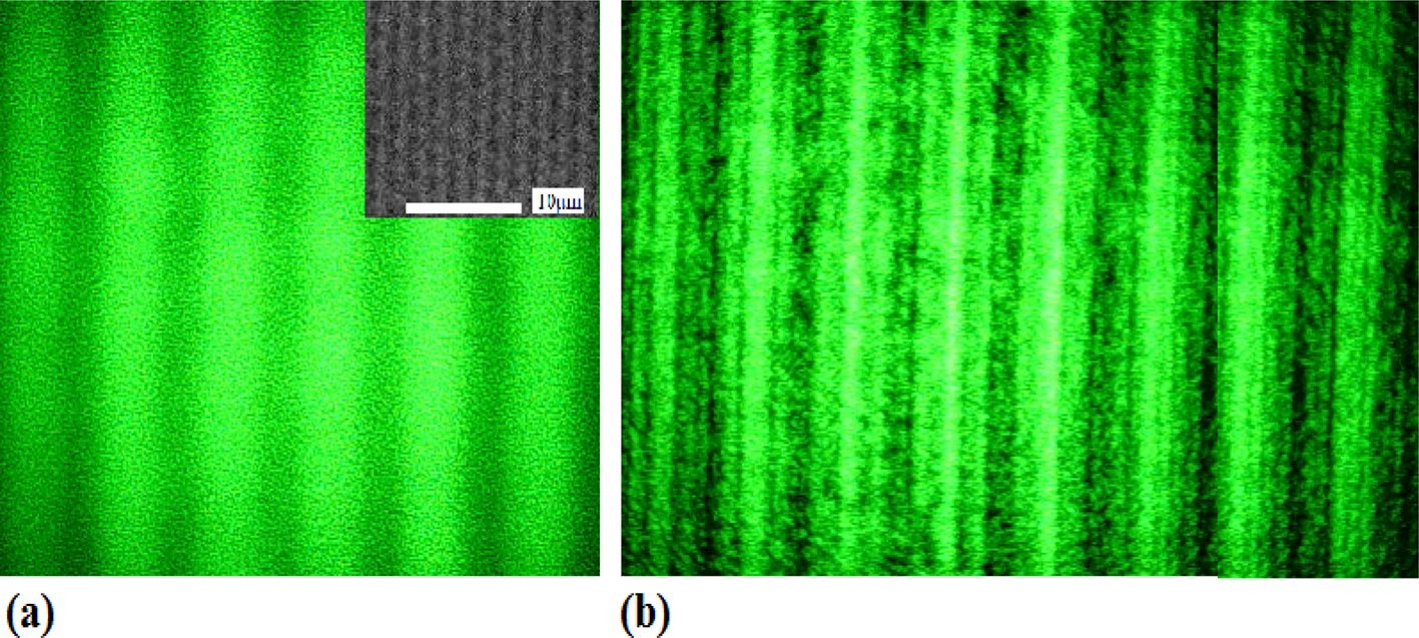
(**a**) General LRTM image, in which the inset is the SEM image; (**b**) nonlinear LRTM image on 1D photonic crystal of silicon.

**Figure 6:**
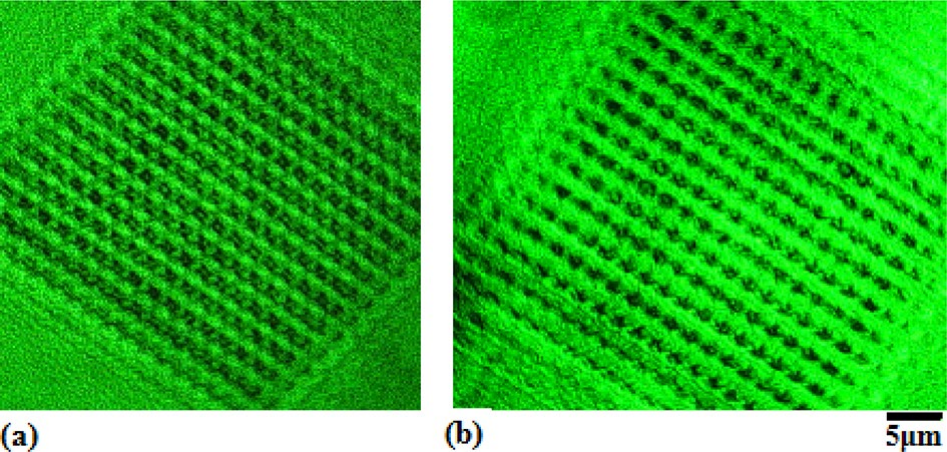
(**a**) General LRTM image and (**b**) nonlinear LRTM image on 2D photonic crystal of silicon.

In the LRTM system with laser at 532 nm, selecting higher-order diffraction and selecting nonlinear reflecting Talbot field can elevate the resolution of image through operating the aperture stop, in which the geometry details related to higher frequencies of space are obtained. Here, the nonlinear LRTM images involve the higher-order diffraction and the higher space frequencies in the reflecting Talbot field. Figures 5 and 6 show the differences between the general reflecting Talbot image and the nonlinear reflecting Talbot image on 1D and 2D photonic crystals of silicon, respectively. Figures 5a and 6a show the general LRTM images on 1D and 2D photonic crystals of silicon, respectively. The inset of Fig. 5a exhibits the SEM image. The higher order diffraction and nonlinear LRTM images on 1D and 2D photonic crystals of silicon are respectively shown in Figs. 5b and 6b, in which the geometry details can be obtained obviously.

It is interesting to make a comparison between Fig. 5a,b, in the latter image there are much more detail geometry structures in the nonlinear LRTM image on the 1D photonic crystals of silicon, which are related to higher order diffraction. In the same way to make a comparison between Fig. 6a,b, the detail geometry structures on the 2D photonic crystals of silicon are exhibited in the nonlinear LRTM image because of involving higher order diffraction.

### LRTM image on plasmonic structures and electronic crystal

The plasma is produced on silicon surface by using nanosecond pulsed laser at 355 nm, in which the plasma frequency is described by the form:

3 $$ \upomega^{2}{_{{\text{p}}}} = {\text{ Ne}}^{{2}} /\upepsilon {\text{m}} $$

where N is electron density of plasma^35^. The plasmonic structures can be built on silicon surface due to resonance by coupling between photon and plasma, where the plasmon frequency is described by the formula:

4 $$ \upomega^{2}{_{{\text{q}}}} =  \upomega^{2}{_{{\text{p}}}} + \, \left( {{3}/{5}} \right){\text{q}}^{{2}} \left( {{\text{hk}}_{{\text{f}}} /{2}\uppi {\text{m}}} \right)^{{2}} $$

n which k_f_ is the Fermi vector and q is the quantum number of Plasmon^36,37^. In the experimental system, the periodic plasmonic structures take place on silicon surface, involving 1D and 2D periodic structures are detected on the Talbot screen, in which Talbot laser and lens are used respectively in the imaging process of the LRTM effect. The LRTM images of the periodic plasmonic structures on silicon surface can be exhibited on the Talbot screen.

**Figure 7:**
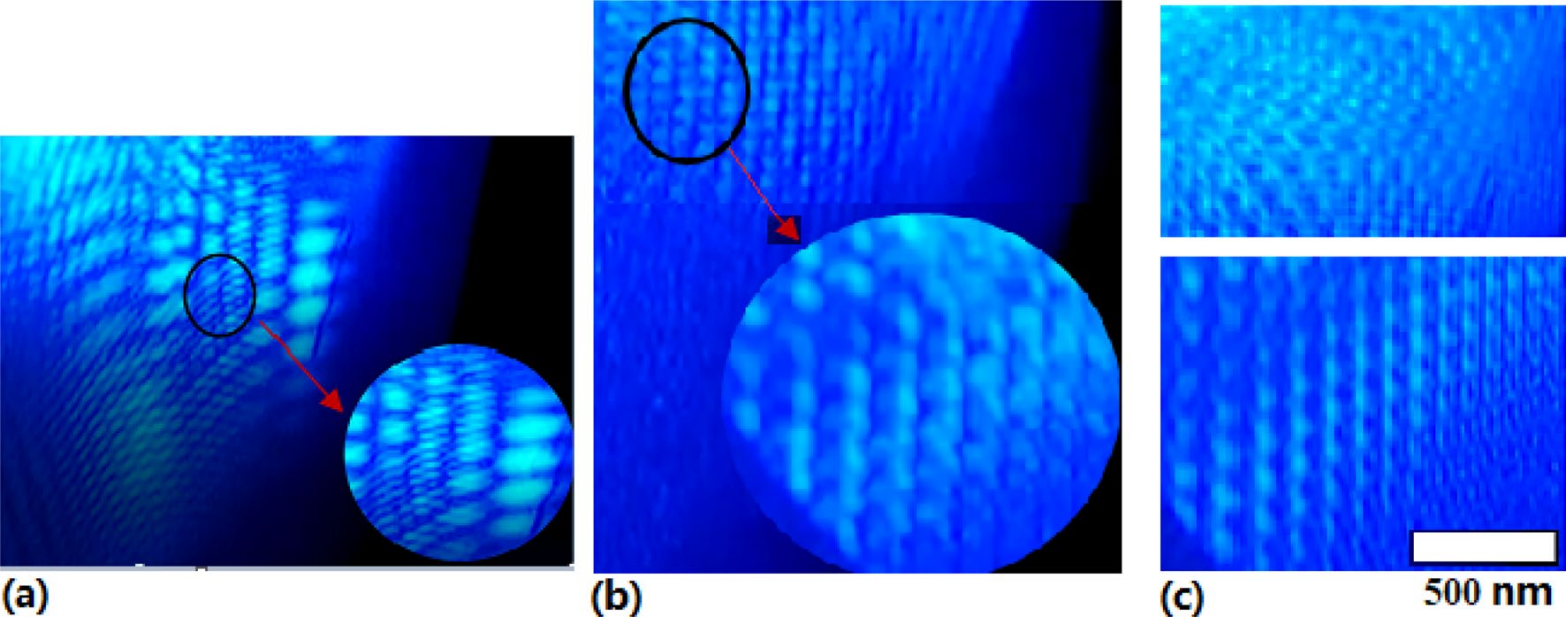
(**a**) LRTM image of the 1D plasmonic periodic structures on silicon surface in the PLE process, (**b**) laser reflecting Talbot magnified image of the 2D plasmonic periodic structures on silicon surface in the PLE process, (**c**) LRTM image of the Wigner electronic crystal with the fourfold (bottom) and the sixfold (top) symmetry lattice on silicon surface in vacuum.

Interestingly, the 1D periodic plasmonic structures are observed on silicon surface in the LRTM image as shown in Fig. 7a, and the 2D periodic plasmonic structures are observed on silicon surface in the LRTM image as shown in Fig. 7b, in which the inset circles exhibit the details of plasmonic structures.

The interaction between photons and plasmons produces surface electron gas on silicon ions background in vacuum with nanosecond pulsed laser irradiation on silicon surface, where only electrons can follow with the photons resonance with high frequency to separate the electrons for generating the electron crystal on surface^36^.

The temporary periodic structure of electronic crystal occurs in vacuum, where reflecting Talbot image demonstrates that the electrons play a main role in the periodic structures on silicon surface in vacuum because of only electronic resonance speeding with photons in nanosecond scale. Figure 7c shows the Wigner electronic crystal on the LRTM image in vacuum, in which the rectangle and hexagonal lattices of transient anomalous electron crystal have been observed on silicon surface at first. These beautiful shapes of the electron crystal structures occur in fourfold and sixfold symmetry respectively, where the size of the electron crystal cells is about 200 nm. It is demonstrated in the decay spectra of the electronic lattice that its lifetime in vacuum is kept in nanosecond scale^38^.

It is interesting to make a comparison between the images of the plasmonic periodic structures and the image of the Wigner electronic crystal, as shown in Fig. 7, where the image (a) and (b) respectively exhibits the 1D and 2D plasmonic periodic structure on silicon surface in Ar gas (10 Pa), but the image (c) shows the Wigner electronic crystal on silicon surface in vacuum (10^–6^ Pa). Here, the differences are very obviously: in the former images, there is not any crystal lattice in the periodic structures, but in the latter image, the two-dimensional Bravais lattices of the electron crystals occur with the fourfold and the sixfold symmetry shapes, where the Wigner electronic crystal with the rectangular lattice (bottom image) and the hexagonal lattice (top image) have obviously been observed on silicon surface in Fig. 7c.

**Figure 8:**
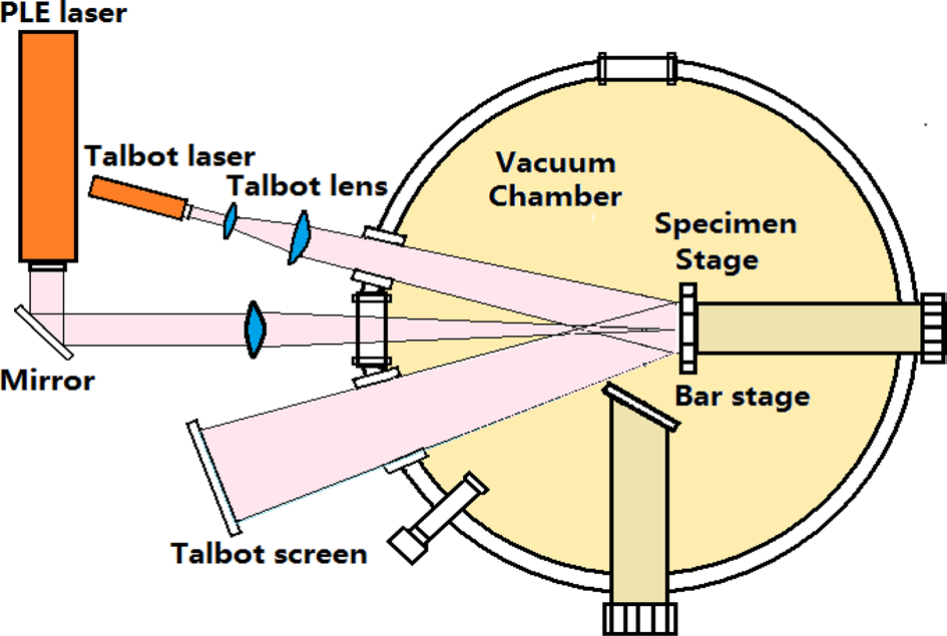
Diagram of the PLE cavity and the LRTM image combination device.

The LRTM effect can be applied in observation on line for the fabrication in the pulsed laser etching (PLE) process, in which the PLE cavity and the LRTM image combination device are shown in Fig. 8.

In the experimental process, a silicon wafer of P-type oriented substrate is taken on the sample stage in the PLE devices, and a third harmonic of pulsed Nd:YAG laser at 355 nm with ns pulsed width is used to etch on Si surface by controlling scanning in vacuum or in different environment. The lattice structure of silicon surface on the specimen stage can be detected on the Talbot screen. In vacuum, the LRTM images of the electron crystal structures can be observed on silicon surface. And the period structures of surface plasmons on the LRTM images in various environments can be detected in the PLE process.

In summary, the LRTM effect under laser illumination has been observed on 1D and 2D photonic crystals of silicon prepared by pulsed laser etching. In the experiment it was demonstrated that the interval of reflecting Talbot images increases with increase of the image distance under laser beam. It is important for application that the magnifying rate of reflecting Talbot images can be controlled by adjusting the wave-front shape for laser illumination. In the LRTM effect, the high-order and nonlinear images have been observed obviously. It should be noted that selecting higher diffraction grades in nonlinear optical process can get a higher resolution in the LRTM images. It is interesting that we have observed the Wigner electronic crystal on plasma surface of silicon by using the LRTM effect image. The various periodic structures of surface plasmons on silicon can be measured in nanosecond pulsed laser etching process through the LRTM effect, which should have a good application on magnifying micro-nanostructures on line of PLE fabrication.

## Method

### Fabrication of 1D and 2D photonic crystals on silicon

Some silicon wafers (100) oriented substrate were taken on the sample stage in the combination fabrication system with pulsed laser etching (PLE) devices. A pulsed Nd:YAG laser (wavelength: 1064 nm, pulse length: 60 ns FWHM, repetition rate: 1000) was used to etch the 1D photonic crystal by line-scanning way and the 2D photonic crystal by point-scanning way on silicon surface. The nanosecond pulsed laser is focused on sample surface, whose spot diameter is in submicron scale. The frame construction of the PLE cavity and the LRTM image combination device is shown in Fig. 8.
